# Unmasking the Veiled Intruder: A Complicated Case of Eosinophilic Fasciitis

**DOI:** 10.7759/cureus.63912

**Published:** 2024-07-05

**Authors:** Asif Uddin, Daniel Lozeau, Asha Patnaik

**Affiliations:** 1 Rheumatology, Allergy, and Immunology, Stony Brook University, Stony Brook, USA; 2 Dermatology, Stony Brook University, Stony Brook, USA

**Keywords:** hypergammaglobulinemia, corticosteroid treatment, monoclonal gammopathy of undetermined significance, sarcoidosis, biopsy, autoimmune disease, paraneoplastic, systemic sclerosis, peripheral eosinophilia, eosinophilic fasciitis

## Abstract

Eosinophilic fasciitis (EF) is a rare inflammatory disease characterized by skin and fascial thickening. Unlike systemic sclerosis, EF lacks internal organ involvement and specific autoantibodies, with peripheral eosinophilia as a hallmark feature. Patients may exhibit joint pain and contractures due to fibrosis. We present a case of a patient who presented with skin thickening involving her upper and lower extremities and was ultimately diagnosed with EF based on a skin biopsy. This case underscores the importance of recognizing the unique clinical and histological features of EF.

## Introduction

Eosinophilic fasciitis (EF) is an inflammatory disease of unknown etiology that affects the skin and its deeper layers. The syndrome was discovered after rheumatologist Lawrence E. Shulman identified two patients with scleroderma-like skin thickening, but with skin biopsies revealing thickening of the fascia rather than changes consistent with scleroderma [1]. Those patients were also found to have peripheral eosinophilia and hypergammaglobulinemia, now known to be important associations of EF. Affected individuals typically present with erythema and edema on the trunk and extremities. As the disease progresses, the edema decreases and the skin becomes thicker and wrinkled, resembling a “peau d’orange” texture. This produces a characteristic phenomenon called the “groove sign,” which refers to visible indentations along the course of superficial veins that develop when an affected extremity is elevated [2]. Patients may also experience pain in their joints due to skin thickening and some develop joint contractures due to advanced fibrosis [3]. The skin and joint manifestations of EF can mimic the findings in systemic sclerosis. However, EF is distinguished by the relative absence of internal organ involvement, the Raynaud phenomenon, and specific autoantibodies. Nailfold capillaroscopy is usually normal in EF. Another distinction is peripheral eosinophilia, which is uncommon in systemic sclerosis but a characteristic feature of EF [4].

## Case presentation

**Figure 1:**
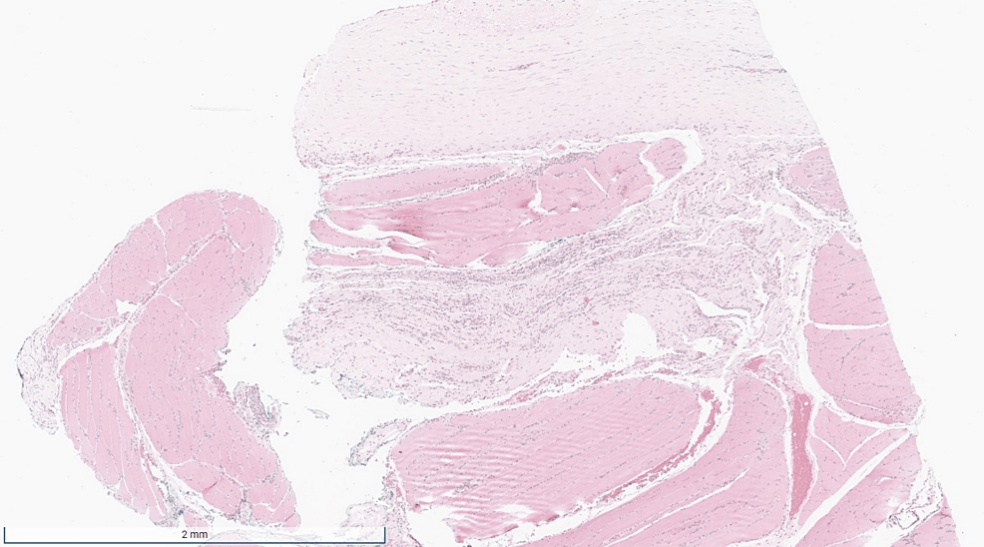
Low magnification reveals a segment of inflamed fascia and skeletal muscle.

**Figure 2:**
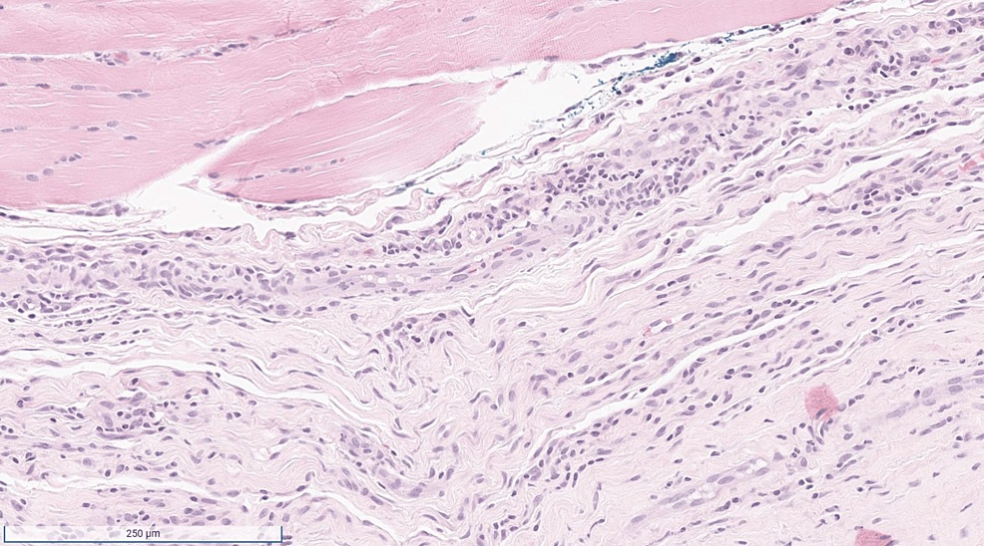
At higher magnification, there is a predominantly lymphocytic inflammatory infiltrate that includes rare eosinophils.

A 58-year-old female presented to the rheumatology clinic for the evaluation of a six-month history of skin thickening involving her bilateral upper and lower extremities. She denied having any joint pains or swelling. She had a previous medical history of monoclonal gammopathy of unknown significance (MGUS), pulmonary nodules, thyroid nodules, hypothyroidism, gastroesophageal reflux disease, and cervical and lumbar degenerative disc disease. In addition, she was diagnosed and treated for multiple infectious diseases in the past year, including herpes zoster, strongyloidiasis, and severe acute respiratory syndrome coronavirus-2 (SARS-CoV-2). She denied smoking cigarettes. She also denied having any known family history of autoimmune diseases. She was taking hydroxyurea and levothyroxine. She saw a hematologist for persistent peripheral eosinophilia despite being treated twice for a suspected parasitic disease. She had undergone a bone marrow biopsy, which revealed a normocellular marrow with an elevated eosinophil count (31%). She subsequently underwent a whole-body PET/CT scan, which revealed hypermetabolic bilateral hilar and mediastinal lymph nodes, and a lymph node biopsy, which reported non-caseating granulomas. Her physical exam was notable for skin thickening involving the extremities distal to the elbows and knees. A “groove sign” was not detected in the forearms. A nailfold capillaroscopy was normal. The results of her laboratory evaluation were notable for an elevated erythrocyte sedimentation rate (ESR) and IgG (Table 1). Laboratory tests for connective tissue diseases were unremarkable. She was referred to a dermatologist and underwent an incisional biopsy involving the fascia in her left forearm that confirmed the diagnosis of eosinophilic fasciitis (Figures 1, 2). The patient was treated with prednisone, which resulted in a decrease in her eosinophil count, but with persistent skin thickening. She was subsequently started on mycophenolate mofetil with the improvement of her skin thickening.

**Table 1:** Laboratory evaluation of the patient. ESR: erythrocyte sedimentation rate; dsDNA: double-stranded deoxyribonucleic acid; SM/RNP: Smith/ribonucleoprotein; SS-A: Sjogren's syndrome A; SS-B: Sjogren's syndrome B; C3: complement component 3; C4: complement component 4; RF: rheumatoid factor; CCP: cyclic citrullinated peptide; IgA: immunoglobulin A; IgG: immunoglobulin G; IgM: immunoglobulin M; WBC: white blood cells.

Variables (units)	Result	Upper limit of normal/range
ESR (mm/hr)	43	2-39
Anti-dsDNA (IU/mL)	<1	≤4
Anti-Smith (units)	<1	<1
Anti-SM/RNP (units)	<1	<1
Anti-SS-A (units)	<1	<1
Anti-SS-B (units)	<1	<1
Anti-Scl-70 (units)	<1	<1
Anti-centromere (units)	<1	<1
C3 (mg/dL)	120	83-193
C4 (mg/dL)	27	15-57
RF (IU/mL)	<10	<10
Anti-CCP (units)	<16	<20
IgA (mg/dL)	357	70-400
IgG (mg/dL)	1820	700-1600
IgM (mg/dL)	117	40-230
WBC (x10^3^/UL)	5.7	4-10.5
Hemoglobin (g/dL)	12.5	11.5-16
Hematocrit (%)	39.7	37-47
Lymphocyte (%)	17.5	20.5-51.1
Eosinophil (%)	1.9	0.7-6%
Eosinophil count (x10^3^/UL)	0.1	0-0.4

## Discussion

Currently, the etiology and incidence of EF remains unknown. Most cases are deemed idiopathic, but EF is associated with infections, medications, autoimmune diseases, and hematologic diseases. Infections with SARS-CoV-2 and vaccinations against it, as well as immunotherapy with checkpoint inhibitors and strenuous exercise, have all been linked to EF as adverse effects [5-8]. The pathophysiology of EF has not been clearly established but is thought to involve activation of interleukin-5 (IL-5) and transforming growth factor-beta (TGF-β) [9]. Establishing the diagnosis can be challenging due to the lack of specific autoantibodies. Therefore, a thorough history and physical exam are needed to exclude other disorders such as systemic sclerosis, which can present similarly to EF. Magnetic resonance imaging (MRI) can reveal thickening and enhancement of the fascial layer as well as subcutaneous and muscle edema [10]. A full-thickness incisional skin biopsy, which assesses the dermis, subcutaneous tissue, and underlying fascia, is currently the preferred procedure to confirm the diagnosis. The characteristic findings on biopsy include fascial thickening with infiltration with lymphocytes, plasma cells, and histiocytes. Eosinophils may or may not be present.

There is currently no single medication that is superior in treating EF. Corticosteroids are commonly used as initial treatment and can lead to a modest improvement of symptoms and a decrease in eosinophilia. A single-center study reported a 94% complete remission in patients who received steroids alone [11]. Other commonly utilized treatments include methotrexate, hydroxychloroquine, mycophenolate mofetil, and azathioprine. Another single-center study reported that 60% of their patients had complete resolution of skin thickening at three years with methotrexate being the most used medication [12]. There are also multiple reports of the interleukin-6 receptor antagonist, tocilizumab, being used in refractory cases with favorable clinical outcomes [13,14]. The targeting of IL-5 using monoclonal antibodies is an intriguing option considering its role in the activation and release of eosinophils [15].

Malignancies including hematologic disorders are an important association of EF. These include aplastic anemia, multiple myeloma, lymphoma, and leukemia. A systematic review found that hematological malignancies can present concurrently or within six months of EF diagnosis, suggesting a paraneoplastic phenomenon [16]. Hypergammaglobulinemia can be present in individuals with EF and warrants close monitoring. Of note, the patient in our case was found to have hypermetabolic hilar and mediastinal lymph nodes, and a biopsy revealed non-caseating granulomas. These findings are not commonly found in EF but are classically seen in sarcoidosis. Our patient did not have any symptoms consistent with sarcoidosis, and her overall presentation was consistent with EF. There are sparse reports describing both EF and sarcoidosis or sarcoidosis-like presentations [17-19].

## Conclusions

In summary, EF is a rare fibrosing disease with clinical features that can sometimes be difficult to distinguish from other diseases such as systemic sclerosis. An inflammatory response is thought to play a role with cytokines that lead to the activation of eosinophils and fibrosis. There is also an association with hematologic disorders such as multiple myeloma and lymphoma. The diagnosis is usually established by excluding mimickers and performing a full-thickness skin biopsy revealing an inflammatory infiltrate in the deep dermis and fascia. Treatment generally consists of corticosteroids and several steroid-sparing agents have been utilized with variable results. Further studies are needed to ascertain the pathogenesis of EF and develop targeted treatment modalities.
